# Moving from *prove* to *improve*: A collaborative continuous quality improvement process for advancing Clinical and Translational Science

**DOI:** 10.1017/cts.2024.555

**Published:** 2024-05-30

**Authors:** Ariel Y. Fishman, David W. Lounsbury, Claudia Lechuga, John Patena, Paul Marantz, Mimi Kim, Marla J. Keller

**Affiliations:** Harold and Muriel Block Institute for Clinical and Translational Research, Albert Einstein College of Medicine and Montefiore Medical Center, Bronx, NY, USA

**Keywords:** Continuous quality improvement, collaborative action planning, common metrics initiative, evaluation, logic modeling

## Abstract

Structured processes to improve the quality and impact of clinical and translational research are a required element of the Clinical and Translational Sciences Awards (CTSA) program and are central to awardees’ strategic management efforts. Quality improvement is often assumed to be an ordinary consequence of evaluation programs, in which standardized metrics are tabulated and reported externally. Yet evaluation programs may not actually be very effective at driving quality improvement: required metrics may lack direct relevance; they lack incentive to improve on areas of relative strength; and the validity of inter-site comparability may be limited. In this article, we describe how we convened leaders at our CTSA hub in an iterative planning process to improve the quality of our CTSA program by intentionally focusing on how data collection activities can primarily advance continuous quality improvement (CQI) rather than strictly serve as evaluative tools. We describe our CQI process, which consists of three key components: (1) Logic models outlining goals and associated mechanisms; (2) relevant metrics to evaluate performance improvement opportunities; and (3) an interconnected and collaborative CQI framework that defines actions and timelines to enhance performance.

## Introduction

Continuous quality improvement (CQI) is a concept in management, health education, and health care delivery through which a group of constituents applies a well-defined methodology to analyze current practices and then implement changes to progress toward a desired performance level [1]. Popular CQI methodologies, such as Lean Management, Six Sigma, Plan-Do-Study-Act (PDSA), and Root Cause Analysis, broadly consist of short cycles of operational change, testing, and evaluation that inform how to sustain long-term improvement in performance, impact, efficiency, and/or extensions to a program’s reach [2,3]. CQI activities help achieve strategic goals by linking near-term actions to long-term organizational performance and sustained equitable access to health care at the population level.

Maximizing quality has been a long-held goal of the National Institutes of Health (NIH) Clinical and Translational Sciences Awards (CTSA) Program, which has recently required that grant awardees have CQI programs. Awardee sites, also known as hubs, must maintain such CQI programs not merely because they fulfill regulatory requirements but also because they are integral to strategic management processes [4]. Yet even though notices of funding opportunities require that CTSA hubs have a CQI program in place, there is no clear guidance as to what quality or efficiency means, which may limit the ability to achieve high-quality programs [5].

In many ways, CQI may be considered a successor to program evaluation processes required in earlier CTSA grant awards. These evaluations aimed to demonstrate that a measurable outcome had been achieved so as “to show that the program is well implemented, efficiently managed, and demonstrably effective [6].” Research Performance Progress Reports (RPPRs), containing standardized data tables about site performance, were submitted with common metrics utilized to *demonstrate* progress since the “lack of high-level common metrics are barriers to overall program *accountability* [emphasis added][7].” Consequent frameworks such as the Common Metrics Initiative (CMI) were established as “a formalized and standardized *evaluation* process” in response to these expectations [8]. These initiatives succeeded in fostering quality improvement in two major respects: (a) they established the benefits of “a formal, structured process for data-driven performance improvement” and (b) they “provided justification […] for devoting the resources and personnel needed for metric-based improvements [9].” CQI has thus generally been framed as a presumed consequence of evaluation activities rather than their primary purpose [10].

A recent survey of hubs noted muted endorsement of CMI’s usefulness in driving quality improvement. Certain metrics were perceived as not relevant [9] due to idiosyncratic differences in operating contexts and variance in data collection approaches, despite the use of objective definitions. Externally reported data could be perceived as potentially biased, and therefore less reliable for comparison purposes, due to concerns about the consequences hubs might face for reporting subpar performance. Survey respondents instead indicated that metrics internally developed by each hub might not only be more relevant in evaluating performance but also more useful in identifying opportunities to improve.

We therefore engaged in a collaborative planning effort to create a CQI process centered around improvement rather than evaluation, with the intention of fostering a deeper, shared understanding of how to work together to achieve and sustain desired impact. This article describes how we convened an iterative planning process to address our hub’s strategic aims and unique goals, seeking to fulfill the CTSA’s requirements to have a CQI program while also integrating CQI as a strategic management tool, cultivating partnership among stakeholders and laying the groundwork for long-term impact.

## Background

**Figure 1.**
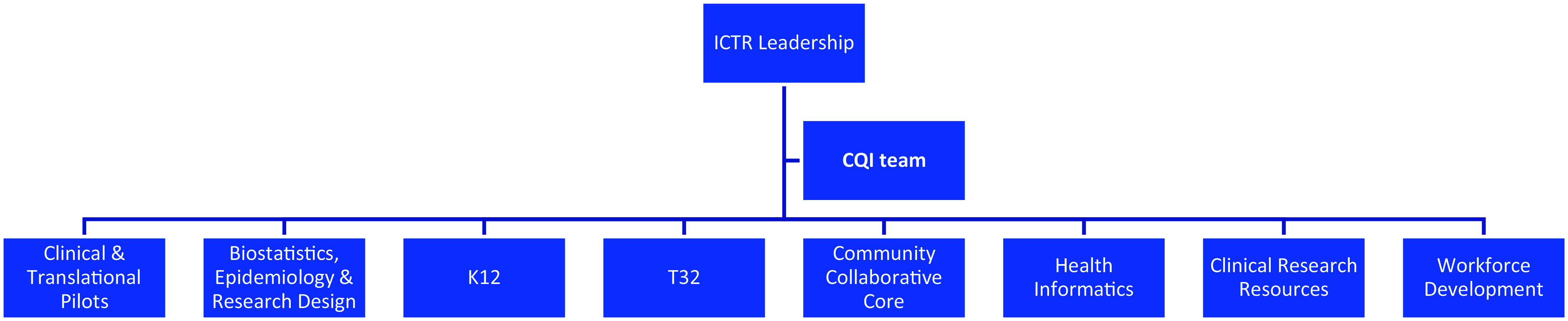
Operating components at Einstein’s Institute for Clinical and Translational Research (ICTR). CQI = continuous quality improvement.

Our CTSA hub is known as the Harold and Muriel Block Institute for Clinical and Translational Research (ICTR), located at Albert Einstein College of Medicine and its partner, the Montefiore Health System. ICTR leadership promotes coordination, communication, and collaboration among the directors of our hub’s eight components (Fig. 1). The CQI team, holding backgrounds in program evaluation and quality improvement, is charged with managing the CQI activities of each component and of the ICTR as a whole.

The CQI team assessed past program evaluation endeavors as part of our most recent CTSA UM1 grant application, awarded in March 2023. Similar to the survey findings of Welch *et al*., [9] we found that internal stakeholders often perceived centralized data collection as primarily serving evaluative or compliance needs, rather than identifying opportunities to advance improvement. Resource-intensive efforts to collect metrics that lacked obvious relevance to components’ goals sometimes distracted directors from considering improvement opportunities in areas not directly evaluated by those metrics. Directors operated under the paradigm that evaluation processes were necessary to demonstrate success and, consequentially, for continued funding. In short: The purpose of data collection was to *prove* rather than *improve* performance.

We sought instead to reframe the purpose of data collection as intentionally advancing CQI rather than strictly serving evaluation or reporting requirements. Our overarching mission was to ensure that CQI functioned to advance the ICTR’s strategic goals: (1) advance translational science, (2) facilitate community and stakeholder engagement, (3) implement scientific resources and services to facilitate clinical and translational research, (4) develop a skilled translational workforce, and (5) partner with other CTSA hubs. Building on learnings gained from the CMI [9], we established protected time for members of the CQI team (about 5%–10% for each of three CQI team members plus administrative support to schedule meetings), recognizing that effective CQI requires institutional resources and commitment. Although we initially had also set aside protected time for a data analyst to support component directors in conducting CQI-based data analysis, we found that directors generally preferred to analyze data on their own.

Throughout the establishment of our CQI process, the CQI team sought to understand the nuances of each component’s goals and center CQI efforts around the component’s needs rather than (solely) on the reporting requirements of the ICTR. We met regularly with each director, emphasizing that they were empowered to select the metrics and tasks needed to achieve progress toward their goals, especially ones that reflected unique characteristics of our hub. We emphasized CQI’s role as part of a broader strategic management process in which ICTR leaders and component directors aligned their activities with one another, supporting rather than supplanting normal managerial decision-making. We encouraged directors to focus on metrics that could facilitate performance improvement irrespective of current levels, permitting (but not centering on) metrics that also happened to serve external reporting requirements such as RPPRs. We downplayed the notion of performance benchmarks and used the same frameworks to discuss performance shortfalls and enhancements alike.

Although we did not explicitly establish a particular theory of change prior to beginning our CQI process, we observed that our efforts implicitly anchored on three premises. First, change sustainably occurs when stakeholders actively participate in the change process, prompting their personal investment in changes and developing their change management skills. As such, we empowered component directors to select CQI projects rather than having ICTR leadership or external stakeholders select where components should engage. Second, change relies on the cultivation of strong relationships and trust among stakeholders, which we built deliberately over time through rapport and an atmosphere of open, honest communication. Third, change can be achieved through small wins that represent milestones toward long-term goals, which enable us to make adjustments based on short-term metrics and provide timely feedback as to whether a change is moving in the right direction.

An overarching summary of our process is framed prescriptively in Table 1. Building on the foundational commitments by our leadership to engage in CQI through appropriate time, personnel, and resource allocation, the CQI team guided directors to articulate their goals and collect appropriate metrics, followed by a repeated, staggered cycle in which progress toward those goals is iteratively advanced and transparently shared through regular convenings and iterations with the CQI team and fellow CQI participants. Instead of being responsible for evaluation, the CQI team was charged with empowering directors to decide where to focus on improvement and how to ensure that it occurred. Our approach ultimately relied on making sure that CQI was genuinely perceived as collaborative, supported, and facilitated rather than externally directed or dictated. We reinforced that CQI was neither evaluative nor punitive, emphasizing that CQI would be used to benefit both underperforming areas and those capable of building on existing success.

**Table 1.** Six steps for implementing a continuous quality improvement (CQI) program

*Establish a foundation:* Commit to engage in CQI though clear support from leadership; a shared desire to improve through collaboration, transparency, and discipline; and sufficient time, personnel, and financial resources to succeed.
*Define goals:* Utilize tools like Logic Models (LMs) to articulate desired impact, measurable goals, and activities that advance those goals. Iteratively revise the LMs to ensure alignment across the organization.
*Collect relevant metrics:* Establish reliable, adaptable, and accessible mechanisms to collect and store data to assess progress toward goals and identify potential areas for improvement.
*Apply a Quality Improvement (QI) framework:* Using a structured framework such as FACE (Focus/Analyze/Change/Evaluate), identify and conduct small-scale improvement projects that advance progress toward long-term goals in ways that are measurable and that can inform future work.
*Continuously engage and cross-pollinate:* Maintain momentum and manage resources by staggering multiple concurrent QI projects; convene leaders at different project phases to share progress, collect feedback, and disseminate lessons learned.
*Assess and adjust:* Ensure that the CQI process itself can be altered using feedback from key stakeholders actively collected across repeated CQI cycles.

## Methods

Our CQI process consists of three essential components: (1) *logic models* outlining goals and corresponding processes; (2) data collection mechanisms and *relevant metrics to evaluate* both actual and hypothesized performance improvement opportunities; and (3) an interconnected *CQI framework* with defined actions and timelines to enhance performance on those metrics.

**Figure 2.**
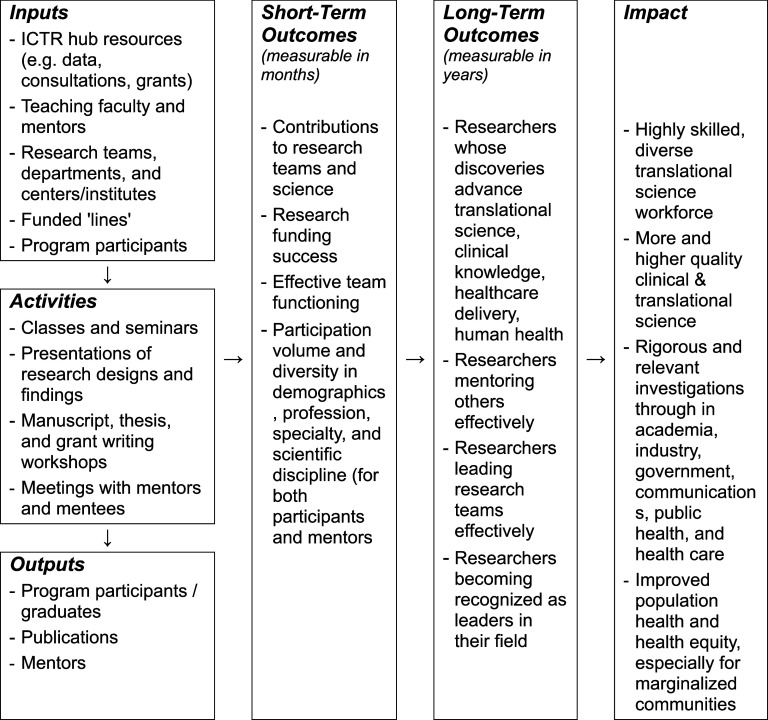
Logic model (sample). Read from left to right; constructed from right to left. ICTR = Institute for Clinical and Translational Research.

Logic models (LMs) are a widely used analytical tool for mapping the relationship between organizational activities and their desired strategic impact [11–13]. Conceptualized and constructed (often) in reverse temporal order, as if reading from right to left, an LM begins by listing areas of intended impact, followed by long-term goals (measurable in years) necessary to achieve that impact and short-term goals (measurable in weeks or months) that demarcate successful progress toward those long-term goals. With goals defined, the LM then articulates the processes (inputs, activities, and outputs) that advance those goals. Read temporally, as if from left to right, a completed LM template illustrates how changes to short-term activities have presumed consequences on long-term outcomes, acknowledging the practical limitations of multi-year metrics in guiding near-term decisions (see Fig. 2). While assertions about causality may lead to questions about the validity of selected measures, as Pincus et al. note, “The challenges of determining causality should not diminish the responsibility of evaluators to discover new methods… for assessing progress toward achievement of CTSA goals [10].”

The CQI team convened with the ICTR leadership and directors to complete LM templates both for the ICTR as a whole and for each component. We collaboratively revised the completed LMs to ensure that activities and goals in each component’s LM were represented in the ICTR-wide LM and vice versa. This process ensured that every activity in a component’s LM related to the goals of the ICTR as a whole and that every goal outlined in the ICTR-wide LM was advanced by at least one component. We also used this LM development process to establish that the role of component directors and ICTR leadership was to define goals, whereas the role of the CQI team was focused on ensuring that those goals were aligned and effectively articulated. Notably, the CQI team also created its own LM to ensure that its activities also aligned with the ICTR’s strategic goals and that the improvement process would itself receive the same attentive focus on quality improvement. We emphasized that LMs were internal tools not used for publication and that they could be revised whenever directors saw fit and as program activities evolved.

With LMs established, the CQI team and directors then collaborated to identify relevant evaluation metrics for each goal. We identified mechanisms and accountable parties for collecting data so that future CQI efforts could leverage data that were collected. Such mechanisms included, but were not limited to, electronic logs of consultation requests, course evaluations and participation rosters, publication databases, and tabulations of grants submitted and funded. Each component director determined the metrics most appropriate for their LM. We encouraged directors to consider the prospective benefits of many metrics, independent of whether they were internally or externally developed. While internally developed metrics were often seen as more relevant, externally developed ones, such as those used in the CMI, can facilitate the sharing of best practices across hubs with varying performance levels.

**Figure 3.**
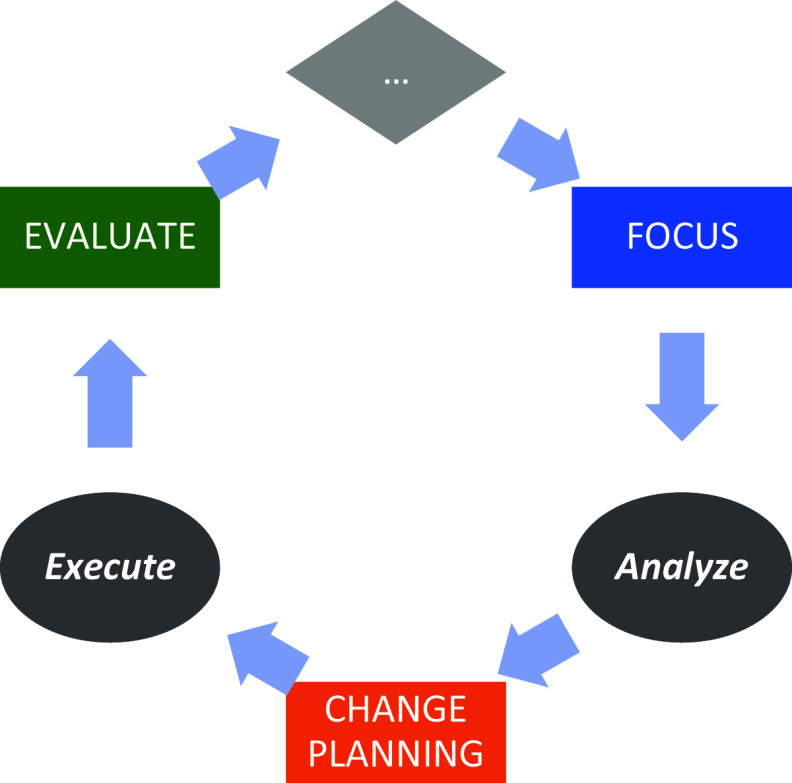
The focus-analyze-change-evaluate cycle.

We then applied a *CQI framework* to advance improvements within each component, repeating the framework on a cyclical basis. We considered the framework through two separate dimensions: “Continuous” and “Quality Improvement.” The Quality Improvement (QI) dimension was manifested by a four-phase paradigm: Focus, Analyze, Change Planning, and Evaluation/Execution, represented by the acronym FACE (see Fig. 3) [14]. We chose this particular paradigm over similar ones like PDSA or Root Cause Analysis, although we believe that the choice of paradigm is less important than the discipline to adhere to a regular CQI process. The acronym FACE was chosen because it reflects the idea that each component should seek to “face” challenges to improve performance.

In the *Focus* phase, directors identify a specific QI project. They begin by generating a list of potential projects, identifying areas that might be falling short of desired goals or otherwise representing an opportunity to improve. Projects have been identified by examining the LM, reflecting on tacit experiences, following up on prior CQI interventions, or through the guidance of end-user stakeholders such as advisory groups. The primary criteria for project selection are (a) that it maps to a goal articulated in the component’s LM, (b) that measurable change can be achieved in less than three months, and (c) that a chain of causality can be asserted that links short-term actions to long-term impact. While ICTR leadership can provide guidance into project selection, the final choice of project rests with the component director, in recognition that the benefits of empowering the director’s decision-making capacity outweigh the consequences associated with an imposed determination of CQI project. If a project is longer than three months in prospective duration, the CQI team helps the director define a near-term milestone to achieve measurable progress and lays the groundwork for continued progress beyond the short-term engagement of the CQI team.

In the *Analysis* phase, directors and CQI team members examine data related to the performance improvement opportunity identified in the Focus phase. These data could be generated from new sources, existing operational activities, external benchmarks, and beyond. The data, which could be quantitative or qualitative, are analyzed to better understand the opportunity, test a specific hypothesis, or explore potential solutions. Data can come from sources such as surveys, enterprise data tabulations, thematic analysis of structured interviews, focus groups, and course evaluation data. The component director, with support from the CQI team if needed, is charged with identifying what data, whether existing or newly collected, can best inform the CQI opportunity, and with directing the analysis most suitable for addressing the area of Focus. The Analysis phase’s goal is to foster data-driven critical thinking and discernment about how to address challenges and opportunities within the identified area of focus.

In the *Change Planning* phase, directors use results of the Analysis phase to explore specific mechanisms to address these challenges and opportunities. Change planning prospectively encompasses activities such as piloting new projects, reallocating resources, investing in newly identified areas of need, or establishing procedural rules to improve fidelity to existing processes. This phase emphasizes engagement in small changes with potentially large impact, accompanied by consideration of their impact on complex, wide-scale changes, especially given that the time associated with change planning is (intentionally) limited to only one month. In some cases, following the Analysis phase, directors reported an emergent recognition that the challenge identified in the Focus phase might be too complex for making small-scale changes, in which cases, they partnered with the CQI team to plan the initial steps for larger-scale change.

In the *Evaluation* phase, directors reflect on their now-executed changes, identifying lessons learned and metrics aligned to assess those changes. This evaluation incorporates short-term reflections (e.g., “What would you have done differently?” and “What lessons would you offer others considering a similar change?”) as well as groundwork for longer-term evaluation (“Did the change achieve its goals?”) that could potentially inform an ensuing project cycle. The relationship between short- and long-term goals, as established in the LMs, can also be adjusted to identify opportunities for potential Focus in future CQI efforts. For projects that underwent pilot efforts during the Change planning phase, a rollout of related initiatives is scheduled. Larger-scale projects could also articulate or affirm timelines for future progress beyond the milestone achieved in the CQI project.

The “continuous” dimension of CQI anchors on the notion that the ICTR engages in multiple FACE projects concurrently, staggering their respective start dates. At any given time, three components are each engaged in a CQI project. The three component directors convene at a standing, monthly meeting with the CQI team, ICTR leadership, and other directors to discuss progress on their projects and share experiences, providing a collaborative forum for directors to present work in progress and solicit constructive feedback.

**Figure 4.**
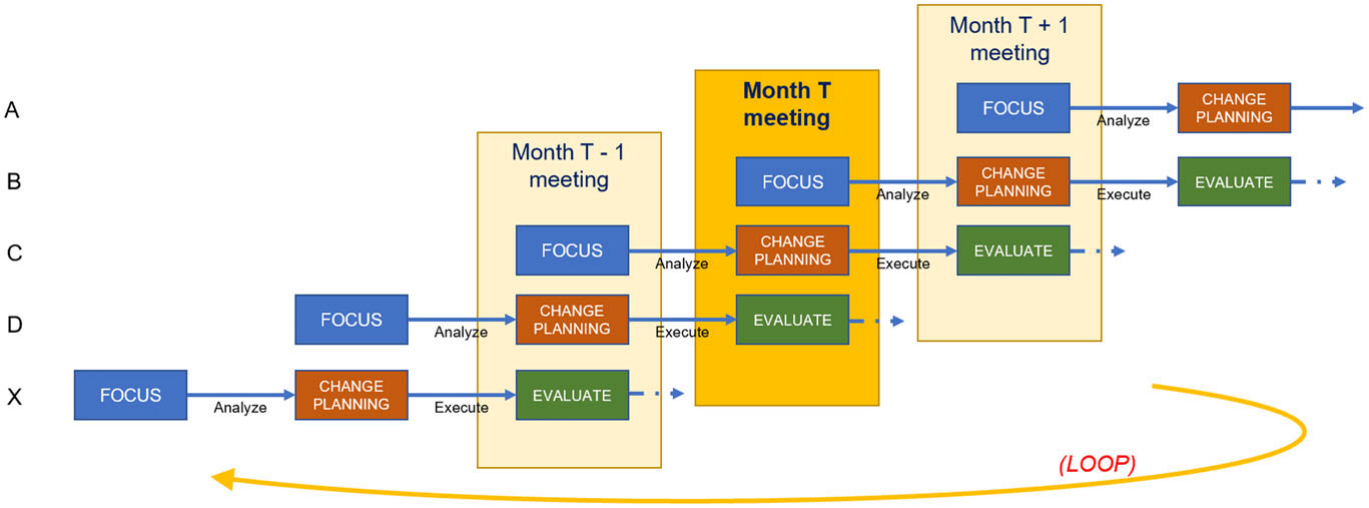
Staggered scheduling across components.

From the directors’ perspective, viewed horizontally in Figure 4, components participation in CQI consists of a three-meeting project cycle. They present their *Focus* project in the first month’s meeting, conduct *Analysis* between the first and second meetings, present their planned *Change* in the second meeting, execute that change between the second and third meetings, and *Evaluate* their lessons learned during the third meeting. Throughout the three-meeting cycle, a designated member of the CQI team provides support for the project. This support may include helping to define the project scope, collecting data, conducting analysis, and acting as a liaison to ICTR leadership. The CQI team member meets with the director as needed, beginning a few weeks before the first meeting and ending with follow-up after the third meeting. A full CQI project cycle therefore spans about three months.

From the CQI team’s perspective, viewed vertically in Figure 4, the structure of a CQI meeting consists of one director presenting their *Focus* decision, a second presenting their *Analysis* findings and *Change* plans, and a third *Evaluating* lessons they have learned. This meeting structure allows directors in later FACE stages to offer guidance to components in earlier stages based on recent experiences. Each director is obligated to attend the three consecutive CQI meetings at which they present, while CQI team members and ICTR leadership hold meetings year-round, helping the CQI team spread its workload. The year-round scheduling of meetings facilitates the continuous nature of CQI as well as the exchange of experiences and ideas across the ICTR. Members of the CQI team convene weekly to share progress among their assigned components and discuss solutions to potential roadblocks.

## Results

As an example of a FACE cycle, the CQI team and the director of a training-related component examined the LM (Fig. 2) and *focused* on data showing that we had received a smaller-than-desired number of applicants for a particular training program. The director hypothesized that the time between initial marketing and application deadlines did not provide enough time for prospective participants to apply to the program. We *analyzed* application deadlines across similar programs and determined that they often required letters of intent (LOIs) from applicants, equipping program directors to reach out to prospective applicants and ensure that they received the support necessary to complete their applications. The implemented *change* involved not only the addition of LOIs but also an adjustment to the overall application calendar, increasing time between marketing launch and application deadlines. With a new calendar in place, the component could eventually *evaluate* whether the quantity and quality of applicants changed compared to prior years.

We have tabulated a list of other ICTR projects in Table 2. At a high level, this list of projects illustrates the wide range of CQI implementations. Project efficacy is intentionally measured by the achievement of short-term goals, representing proximal measures for long-term ones that are yet to occur. As diverse as the projects are in their targeted focus, chosen data methodologies, changes developed, and lessons learned, they reflect a common approach to the advancement of incremental change.

**Table 2.** Selected continuous quality improvement projects at the Institute for Clinical and Translational Research

Component	Timing	Focus	Analyze	Change executed	Evaluation (Long Term)
Workforce Development	May to Jul	Knowledge acquisition	Interviews and focus groups	Collect real-time feedback from learners	Increased learning
Clinical Research Resources	Jun to Aug	Use of training tool	Survey, web analytics	Targeted advertising	Increase tool utilization
Health Informatics	Jul to Sept	Database functionality	User experience analysis	Prioritize development and timing of features	Improve project management process
Community Collaboration	Aug to Oct	Process for prioritizing offerings	Tabulate service requests by type	Develop algorithm and roadmap	Sustain/grow partnerships
Postdoctoral training (K12)	Sept to Nov	Increase diversity	Focus groups with current enrollees	Make past applications available to applicants through web portal	Improve quantity & quality of applications
Predoctoral training (T32)	Oct to Jan	Increase enrollment volume	Focus groups and Interviews	Develop plan to change curriculum	Evaluate new curriculum
Biostatistics, Epidemiology and Research Design	Nov to Feb	Web site functionality	Web site utilization patterns	Overhaul website, add analytics tools	Increase traffic to new site
Translational Science Pilots	Jan to Mar	Educate applicant pool about translational science	Trends in responsive (vs non-responsive) applications	Require pre-submission consultations or workshop participation	Improve applicant volume and quality

The CQI team also conducted a FACE cycle internally. In the team’s LM, we articulated that our long-term goal was to have an effective CQI process, measurable by a short-term goal that component directors express high levels of satisfaction on feedback surveys about the CQI team. We therefore administer a survey after every FACE meeting, making adjustments from time to time based on feedback collected from that survey. Tangible change has included extending FACE meeting invitations to administrative staff, announcing upcoming CQI cycle dates far in advance, and piloting expansion of the time between meetings to enable directors to engage in projects requiring more in-depth analysis or time to execute change.

The key feature of our CQI program has been the iterative convening of the CQI team, ICTR leadership, and component directors, staggered among components at different phases of a CQI project. Through these meetings, we not only harness the collective input and resources of the ICTR to address improvement opportunities but also foster a culture of partnership between the CQI team and component directors, enhancing the local relevance and impact of the projects selected. Because the relationship between short- and long-term goals is established in the LM, the relatively rapid, three-month CQI process for improving short-term goals has had the intended consequence of progressing toward long-term goals.

## Discussion

We designed the CQI process to be collaborative, iterative, and with regular touchpoints. Directors indicated that having accountability to present CQI projects in process helped them move CQI forward within their components by establishing meaningful deadlines and catalyzing internal staff. Leadership’s emphasis on CQI’s role as centered around improvement, rather than strictly evaluation, has helped directors select projects they felt would best help them advance their goals. Concurrent presentation of CQI projects (see Fig. 4) has created a mechanism for directors and staff from across the ICTR to cross-pollinate ideas, collaborate on joint projects across components, build trusting rapport (relational capacity), and work together to achieve complementary goals and objectives.

As directors gained more experience with CQI, the CQI team reinforced norms such as ensuring that discussions were to be considered informal, collaborative, and constructive, and that partially developed ideas were welcome because they fostered healthy discussion. Both at one-on-one and at FACE meetings, the CQI team emphasized that their role was to facilitate, rather than evaluate or audit, and that performance at any level could be improved. Directors were encouraged to use CQI team members as a resource to help collect or analyze data, calibrate project size, or leverage their familiarity with projects conducted elsewhere within the ICTR. The most substantive challenges to the CQI process were generally logistical: maintaining the discipline to schedule meetings, keeping to an agenda, and maximizing attendance despite the challenging schedules of busy research leaders. As we continue to improve our CQI process, we aim to assess how participation in successive FACE cycles has enhanced relational capacity and nurtured new partnerships within our ICTR and beyond.

We acknowledge that our approach is oriented around component directors being able to select their own CQI efforts (bottom-up) instead of having CQI directed by ICTR leadership (top-down). We felt that a top-down approach could bear too much resemblance to an evaluative, “prove”-based mindset that could feel disempowering to directors and could risk creating a process centered on metrics with little local relevance. Rather than holding directors accountable for outcomes that may ultimately be out of their control, our process holds directors accountable for engaging in CQI as a process, trusting their expertise in identifying goals, enabling them to openly discuss challenges, developing their skills, and ultimately achieving an “improve”-based mindset in which improvement activities are intrinsically motivated.

## Conclusion

While we do not contend that the CQI process implemented at the ICTR is an entirely novel concept, we have sought to demonstrate how the fundamental components of established CQI approaches can be applied to advancing the quality of clinical and translational research. We suggest that CTSA hubs and other institutions seeking to advance clinical and translation science can improve by incorporating LMs and a CQI paradigm such as FACE into their strategic management processes. Framing CQI as a paradigm shift from an evaluation-centric to an improvement-centric approach may catalyze greater success.

As we continue to implement the CQI process within each ICTR component, we intend to revisit how our ICTR-wide LM might incorporate a more nuanced focus on translational science in addition to translational research, reflecting a CTSA consortium-wide shift toward translational science and its associated metrics. As more hubs engage in CQI processes, we aim to explore how to leverage cross-hub collaboration to improve not only our own performance but that of other hubs as well. We also have come to recognize opportunities to apply this CQI approach in creative ways, such as by catalyzing opportunities for inter-component collaboration on matters for which they share key goals or operational activities. We highlight that it is the process of working with teams at all levels in a collaborative and iterative way that makes the most impact.
